# Association between maternal intimate partner violence and health-related quality of life in their preschool children: The mediating role of maternal parenting styles

**DOI:** 10.3389/fpsyt.2022.996971

**Published:** 2022-11-08

**Authors:** Shengyu Luo, Li Lin, Weiqing Chen, Chunrong Li, Yan Ren, Meng Zhang, Vivian Yawei Guo

**Affiliations:** ^1^Department of Epidemiology, School of Public Health, Sun Yat-sen University, Guangzhou, China; ^2^Chengdu Women’s and Children’s Central Hospital, School of Medicine, University of Electronic Science and Technology of China, Chengdu, China

**Keywords:** intimate partner violence, health-related quality of life, parenting styles, preschool children, mediation

## Abstract

**Background:**

Although intimate partner violence (IPV) against women is a public health issue around the world, there is a lack of evidence regarding the impact of maternal IPV on preschool children’s health-related quality of life (HRQOL). Therefore, the aim of this study was to investigate the association between maternal IPV and HRQOL among Chinese preschool children, as well as the mediating role of maternal parenting styles.

**Methods:**

A cross-sectional study was conducted with 4,243 mother-child dyads who attended preschools. Mothers self-reported their parenting styles and experience of IPV. Children’s HRQOL was collected through mother-proxy report with the Pediatric Quality of Life Inventory version 4.0 (PedsQL 4.0). Multivariate linear regression analysis was conducted to evaluate the association between maternal IPV and children’s HRQOL. Mediation models were further applied to explore the possible mediating role of maternal parenting styles.

**Results:**

Of the included mothers, 7.4% had experience of IPV. Compared to children of mothers without any IPV exposure, those of mothers with experience of IPV had significantly lower scores in all HRQOL dimensions and summary scales. After adjustment for covariates, maternal IPV was significantly associated with children’s lower physical health summary score, psychosocial health summary score, and total scale score. Mediation analysis showed that both rejection and overprotection mediated such associations, but not for the emotional warmth.

**Conclusion:**

Our findings indicated the need to screen maternal IPV supplemented with targeted interventions focusing on parenting styles, in order to mitigate the negative impact of maternal IPV on children’s HRQOL.

## Introduction

Intimate partner violence (IPV) against women is prevalent around the world. In 2018, the estimated global prevalence of physical and/or sexual IPV was 27% among women aged between 15 and 49 years (1). In regard to the situation in China, a review has summarized relevant studies and found that the lifetime prevalence ranged from 24.5 to 30.0% for psychological IPV, and from 5.4 to 34.0% for physical IPV in Chinese women (2). A recent study conducted in 6 provinces across China has even shown that the lifetime prevalence of psychological and physical IPV was as high as 77.7 and 40.2% in female adults, respectively (3). The high prevalence of IPV in China makes it a public health issue with widespread concern.

It is well-accepted that women who had experienced IPV were at higher risk of both mental and physical impairment, as well as premature death (4–7). A large body of evidence has further revealed that the detrimental impact of IPV against mothers could also extend to the next generation, leading to delayed development, poorer physical health, and more psychosocial problems in their children (8–10). For example, a cross-sectional study conducted in South Africa has found that children with maternal report of IPV were more likely to fall behind their peers in cognitive and language development (8). Longitudinal studies have also suggested that children of mothers with IPV exposure were at higher risk of both physical and psychological health problems, including asthma, and posttraumatic stress disorder (9, 10). In addition to above-mentioned health issues, maternal IPV could also impair children’s health-related quality of life (HRQOL), a multidimensional construct that includes physical, psychological, and social wellbeing (11). A small-scale study conducted in China has shown a significant link between maternal IPV and children’s poorer psychosocial HRQOL (12). Another cross-sectional study conducted in Japan has also found that children with mothers of IPV exposure tended to have poorer HRQOL (13).

The mechanisms of the association between maternal IPV and poorer HRQOL in offspring remain unclear. One of the possible explanations is related to parenting styles, an multifaced and complex family factor that could affect children’s physical and psychosocial development (14). Parents rear and encourage their offspring to learn to inhibit actions that may be harmful or annoying to others, and meanwhile, to acquire behaviors that the society and culture demand, such as self-reliance, consideration for others, accepting responsibility, and skills that will help them to become competent members of the society, which is called parental socialization (15, 16). One of the seminal theories of parental socialization is the tripartite model “Y” proposed by Baumrind (17). This model consisted of three parenting styles (i.e., authoritative, authoritarian, and permissive) based on the interaction between affection, communication, and control, yielding three modes of parental control, including the authoritative control, the authoritarian control, and the permissive control (i.e., lack of control) (17, 18). Furthermore, Maccoby and Martin have proposed a bidimensional model of warmth and strictness, which has become the referential model for studying parental socialization (15, 16). Parental warmth refers to how parents support and communicate with their children and show care and acceptance to them (19–21). It has been labeled with other names with similar meanings, such as acceptance/involvement, assurance, nurturance or love (16, 19, 22–25). In contrast, the dimension of parental strictness was defined as how parents use control and supervision to uphold their authority, moderate children’s behaviors, and establish norms for them (22, 26). Domination, hostility, inflexibility, firm control, or restriction are some of the labels applied in family studies to indicate strictness dimension (16, 20, 22, 25, 27). Based on these two dimensions, four different parenting styles were identified: authoritative parents (high in both warmth and strictness), authoritarian parents (low in warmth and high in strictness), indulgent parents (high in warmth and low in strictness), and neglectful parents (low in both warmth and strictness) (15, 16). Previous studies have shown that mothers with experience of IPV were more likely to adopt parenting styles of neglect, psychological aggression, and physical aggression (28, 29). For example, a review of 136 studies has illustrated that mothers who suffered from IPV tended to engage in harsh, rejective, and overprotective parenting styles (29). Furthermore, maternal rejection has been demonstrated to be associated with poorer HRQOL in children (30). Therefore, parenting styles might play a role in the association between maternal IPV and children’s HRQOL.

In spite of the aforementioned findings, few studies have explored the associations of maternal IPV, children’s HRQOL, and maternal parenting styles simultaneously. Thus, the current cross-sectional study aims to (1) evaluate the association between maternal IPV and HRQOL of their children attending preschools, and (2) explore whether maternal parenting styles mediate such associations.

## Materials and methods

### Sample and procedure

This cross-sectional study was conducted in Chengdu, a megacity with 12 urban districts, 5 county-level cities, and 3 counties in western China. To select representative children from preschools, a multistage sampling strategy was applied. In the first stage, 4 urban districts, 2 county-level cities, and 1 county were randomly selected. In the second stage, two preschools were further randomly chosen from each selected area, with 14 preschools in total. In the last stage, all children in the selected preschools and their parents were invited to join the study. From May to July 2021, caregivers of 5,102 preschool children have finished an online questionnaire (response rate: 86.5%). We have further excluded 795 children as the questionnaires were answered by their fathers, 23 children with answers from their grandparents or other caregivers and 41 children with misreport of age, leaving 4,243 mother-child dyads in the current analysis.

Ethical approval for the study was obtained from Sun Yat-sen University prior to participation (Reference number: 2021[116]), and informed consent was obtained from each parent.

### Measures

In consistent with previous studies (31, 32), maternal IPV status was assessed by two questions: (1) Have you ever been psychologically hurt by your intimate partner (e.g., scolding, belittling, humiliating, and intimidation)? (2) Have you ever been physically hurt by your intimate partner (e.g., slapping, hitting, kicking, and beating)? Answers to each of these questions were “yes” or “no.” A mother was considered as a victim of IPV if she has reported a positive answer (i.e., yes) to either of the question.

Children’s HRQOL was reported by their mothers using the Pediatric Quality of Life Inventory Version 4.0 (PedsQL 4.0), which has been validated in Chinese population (33). The questionnaire consists of 21 items for young children aged between 2 and 4 years and 23 items for those aged between 5 and 7 years. Both versions are comprised of four subscales, including physical functioning (8 items, e.g., “lifting something heavy”), emotional functioning (5 items, e.g., “feeling sad”), social functioning (5 items, e.g., “getting teased by other children”), and school functioning (3 items for 2–4 years old children, and 5 items for 5–7 years old children, e.g., “missing school/daycare because of not feeling well”) (34). Each item is rated on a 5-point Likert scale (0 = never a problem, 1 = almost never a problem, 2 = sometimes a problem, 3 = often a problem, and 4 = almost always a problem), and reversely transformed to 0∼100 (0 = 100, 1 = 75, 2 = 50, 3 = 25, and 4 = 0). The average score of all items in each subscale is used to assess children’s HRQOL in different dimensions, with a higher score indicating better HRQOL. In addition, three summary scores were further generated, namely physical health summary score, psychosocial health summary score, and total scale score. The physical health summary score was represented by the physical functioning dimension, while the psychosocial health summary score was calculated as the average score of the emotional functioning, social functioning, and school functioning dimensions. A total scale score of the overall HRQOL was further calculated as the average score of all items in the four dimensions.

Maternal parenting styles were measured by the short Egna Minnen Beträffande Uppfostran Parent Form (S-EMBU-P), a 21-item questionnaire to assess self-reported parenting styles with three dimensions, including emotional warmth (7 items, e.g., “I praise my child”), rejection (6 items, e.g., “I get angry with my child without letting him/her know the reason”), and overprotection (8 items, e.g., “My child wishes I would worry less about what he/she is doing”) (35, 36). The emotional warmth dimension refers to the degree that parents praise, approve, encourage, help, and show affection to their children, while the rejection dimension and the overprotection dimension describe the degree to which parents direct, command, punish, and impose rules and restrictions to their children (37). All items are rated on a 4-point Likert scale (1 = never, 2 = seldom, 3 = often, and 4 = most of the time). The score of each parenting style was calculated by adding up the items in the corresponding dimension and a higher score indicates more emotional warmth, more rejective behaviors, and more overprotection from mothers, respectively.

Children’s age, gender, status of single child, sleep duration, and primary caregiver were reported by their mothers. The status of single child was classified as yes (with one child in the family) or no (with more than one child in the family). Children’s sleep duration was calculated as (5 × sleep duration on weekdays + 2 × sleep duration on weekend)/7, and was further separated into < 10 h/day and ≥ 10 h/day groups. Primary caregivers of children were categorized as mothers, fathers, and others.

Mothers self-reported their age, current marital status, educational level, and monthly per-capita income. Current marital status was separated into married and unmarried groups. The latter included single, divorced, separated, and widowed. Educational level was grouped into (1) junior high school or below, (2) senior high school, (3) bachelor’s degree, and (4) master’s degree and above. Monthly per-capita income was categorized as (1) ≤ 5,000 RMB, (2) 5,001∼10,000 RMB, (3) 10,001∼15,000 RMB, (4) > 15,000 RMB per month, and (5) uncertain, where 1US$≈ 7.2 RMB.

### Analysis plan

Descriptive statistics were reported as mean (*SD*) for continuous variables and frequencies (%) for categorical variables. Characteristics of children and their mothers between different maternal IPV groups were compared with independent student’s *t*-tests for continuous variables and Chi-square tests for categorical variables.

Pearson’s correlation coefficients were applied to examine the correlation between four subscales of HRQOL (i.e., physical functioning, emotional functioning, social functioning, and school functioning) and three dimensions of parenting styles (i.e., emotional warmth, rejection, and over protection).

Multivariate linear regression was conducted to assess the association between maternal IPV and children’s HRQOL with adjustment for children’s age, gender, status of single child, sleep duration, and primary caregiver, as well as maternal age, current marital status, educational level, and monthly per-capita income. For all linear regression models, assumptions of linear regression models, including linearity, normality, homoscedasticity, and absence of multicollinearity were checked.

The mediating effect of maternal parenting styles in the association between maternal IPV and children’s HRQOL was examined by the mediation model in PROCESS 3.3 invented by Preacher and Hayes (38). The 95% confidence intervals (CIs) were estimated using 5000 bootstrap samples to test the significance of mediating effects. The model was adjusted for the same covariates mentioned above.

All data were analyzed using SPSS version 26.0 and a *p*-value < 0.05 (two-sided) was considered as statistically significant.

## Results

**FIGURE 1:**
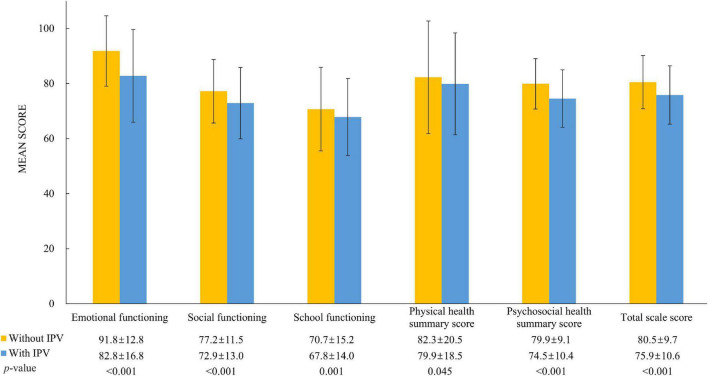
Comparison of children’s HRQOL by maternal IPV status using independent student’s *t*-tests. IPV, intimate partner violence; HRQOL, health-related quality of life.

Of the 4,243 mother-child dyads included in the current study, the mean age was 33.1 (*SD*: 4.6) years for mothers and 4.6 (*SD*: 1.0) years for children (Table 1). Approximately 7.4% (*n* = 316) mothers had experience of IPV. Among them, 304 have experienced psychological IPV, 86 have experienced physical IPV, and 74 have experienced both types of IPV in their lifetime. Compared to those without any IPV exposure, mothers with experience of IPV were relatively older and more likely to be unmarried. In addition, mothers with IPV exposure were more likely to conduct parenting styles of rejection and overprotection, compared to their counterparts who were never exposed to any kind of IPV. In terms of the comparison of children’s characteristics by maternal IPV status, we found that compared to children of mothers without experience of IPV, those of mothers with IPV exposure were more likely to be girls and had shorter sleep duration. Furthermore, children of mothers with IPV exposure had significantly lower HRQOL scores in all dimensions and summary scales, compared to those of children whose mothers had not reported IPV (Figure 1).

**TABLE 1:** Comparison of characteristics of children and mothers by maternal IPV status.

	Mother-child dyads			
	Total	With IPV	Without IPV	*P-value*
*N*, (*n*%)	4,243	316 (7.4)	3,927 (92.6)	
**Maternal characteristics**				
Maternal age (year), mean (*SD*)	33.1 (4.6)	33.7 (4.7)	33.1 (4.6)	0.025
Current marital status, *n* (%)				<0.001
Married	4,090 (96.4)	268 (84.8)	3,822 (97.3)	
Unmarried^a^	153 (3.6)	48 (15.2)	105 (2.7)	
Educational level, *n* (%)				0.300
Junior high school or below	387 (9.1)	29 (9.2)	358 (9.1)	
Senior high school	988 (23.3)	64 (20.3)	924 (23.6)	
Bachelor’s degree	2,635 (62.2)	200 (63.3)	2,435 (62.1)	
Master’s degree or above	229 (5.4)	23 (7.3)	206 (5.3)	
Monthly per-capita income, *n* (%)				0.400
≤ 5,000 RMB^b^	1,174 (27.7)	98 (31.0)	1,076 (27.4)	
5,001∼10,000 RMB	1,107 (26.1)	83 (26.3)	1,024 (26.1)	
10,001–15,000 RMB	634 (14.9)	48 (15.2)	586 (14.9)	
> 15,000 RMB	795 (18.7)	57 (18.0)	738 (18.8)	
Uncertain	533 (12.6)	30 (9.5)	503 (12.8)	
**Parenting styles (measured by S-EMBU-P, mean (*SD*)**				
Emotional warmth	22.8 (4.5)	22.4 (4.5)	22.8 (4.5)	0.147
Rejection	7.6 (1.7)	8.3 (1.9)	7.5 (1.7)	<0.001
Overprotection	15.6 (2.8)	16.1 (2.8)	15.6 (2.8)	<0.001
**Children’s characteristics**				
Age (years), mean (*SD*)	4.6 (1.0)	4.6 (1.0)	4.6 (1.0)	0.964
Gender, *n* (%)				0.013
Boy	2,193 (51.7)	142 (44.9)	2,051 (52.2)	
Girl	2,050 (48.3)	174 (55.1)	1,876 (47.8)	
Status of single child, *n* (%)				0.120
Yes	1,957 (46.1)	159 (50.3)	1,798 (45.8)	
No	2,286 (53.9)	157 (49.7)	2,129 (54.2)	
Sleep duration, *n* (%)				0.001
≥ 10 h/day	2,436 (57.4)	152 (48.1)	2,284 (58.2)	
< 10 h/day	1,807 (42.6)	164 (51.9)	1,643 (41.8)	
Primary caregiver, *n* (%)				0.192
Mother	3,137 (73.9)	225 (71.2)	2,912 (74.2)	
Father	148 (3.5)	8 (2.5)	140 (3.6)	
Others	958 (22.6)	83 (26.3)	875 (22.3)	

SD, standard deviation; IPV, intimate partner violence; S-EMBU-P, the short Egna Minnen Beträffande Uppfostran Parent Form. ^a^Unmarried included single, separated, divorced, and widowed; ^b^1 US $≈7.2 RMB.

The correlation matrix between children’s HRQOL and maternal parenting styles is presented in Table 2. There was a significant correlation between maternal emotional warmth and higher scores in all subscales of children’s HRQOL, except physical functioning. In the contrary, maternal rejection and overprotection were negatively correlated with all subscales of children’s HRQOL.

**TABLE 2:** Correlation matrix between children’s HRQOL and maternal parenting styles.

	Emotional warmth	Rejection	Over protection	Physical functioning	Emotional functioning	Social functioning	School functioning
Emotional warmth	1.000						
Rejection	−0.141***	1.000					
Over protection	0.200***	0.390***	1.000				
Physical functioning	0.007	−0.189***	−0.142***	1.000			
Emotional functioning	0.031*	−0.295***	−0.162***	0.240***	1.000		
Social functioning	0.128***	−0.252***	−0.126***	0.407***	0.360***	1.000	
School functioning	0.284***	−0.183***	−0.074***	0.025	0.126***	0.231***	1.000

HRQOL, health-related quality of life. *p < 0.05, ***p < 0.001.

**FIGURE 2:**
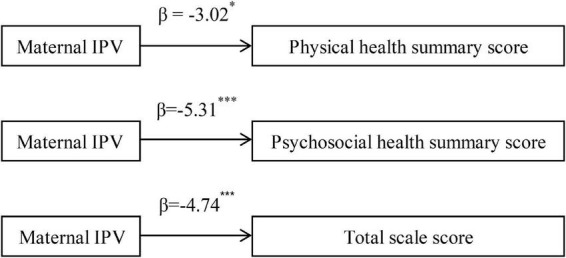
Association between maternal IPV and summary scores of children’s HRQOL. IPV, intimate partner violence; HRQOL, health-related quality of life. (Adjusted for children’s age, gender, status of single child, sleep duration, primary caregiver, maternal age, current marital status, educational level, and monthly per-capita income). *p < 0.05, ^***^p < 0.001.

Figure 2 shows the associations between maternal IPV and summary scores of children’s HRQOL. The results showed that maternal IPV was significantly associated with lower physical health summary score (β = –3.02, *p* < 0.05), psychosocial health summary score (β = –5.31, *p* < 0.001), and total scale score (β = –4.74, *p* < 0.001) after adjustment for covariates.

**FIGURE 3:**
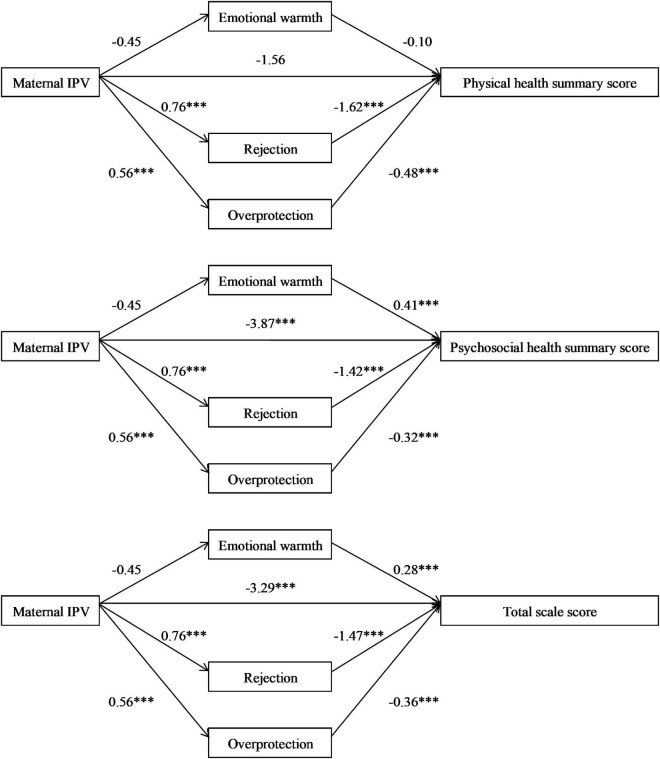
Mediating effect of parenting styles between maternal IPV and summary scores of children’s HRQOL. IPV, intimate partner violence; HRQOL, health-related quality of life. (Adjusted for children’s age, gender, status of single child, sleep duration, primary caregiver, maternal age, current marital status, educational level, and monthly per-capita income). ^***^p < 0.001.

Figure 3 presents the results of mediation analysis. Mothers with experience of IPV were more likely to be rejective (β = 0.76, *p* < 0.001) and overprotective (β = 0.56, *p* < 0.001) toward their children. They also tended to have lower scores on self-rated emotional warmth, but with borderline significance (β = –0.45, *p* = 0.078). Furthermore, maternal emotional warmth was significantly associated with higher psychosocial health summary score (β = 0.41, *p* < 0.001) and total scale score (β = 0.28, *p* < 0.001), but not for the physical health summary score (β = –0.10, *p* = 0.161). In contrast, both rejection and overprotection were significantly associated with lower physical health summary score, psychosocial health summary score, and total scale score. After introducing parenting styles into the model, the associations of maternal IPV with psychosocial health summary score (β = –3.87, *p* < 0.001) and total scale score (β = –3.29, *p* < 0.001) were decreased but remained significant. However, the direct impact of maternal IPV on children’s physical health summary score was not statistically significant anymore after adjustment for parenting styles.

Further mediation analyses indicated that parenting styles was a significant mediator in the association between maternal IPV and poorer HRQOL in offspring, attributable to 48.3, 27.3, and 30.6% of the total effect in the association of maternal IPV with physical health summary score, psychosocial health summary score, and total scale score, respectively. Specifically, the indirect effects of rejection and overprotection were significant in all three models. However, the mediating effect of emotional warmth was not significant in any of the models (Table 3).

**TABLE 3:** Mediating effect of parenting styles between maternal IPV and summary scores of children’s HRQOL.

	Point estimate	Bootstrap 95%CI		Ratio of effect^a^
		Lower	Upper	
**Maternal IPV on physical health summary score**				
Total	–3.02*	–5.33	–0.71	
Direct	–1.56	–3.85	0.72	51.7%
Indirect				
Total	–1.46*	–2.00	–0.96	48.3%
Emotional warmth	0.05	–0.02	0.16	
Rejection	–1.23*	–1.74	–0.81	
Overprotection	–0.27*	–0.50	–0.09	
**Maternal IPV on psychosocial health summary score**				
Total	–5.31*	–6.38	–4.24	
Direct	–3.86*	–4.87	–2.86	72.7%
Indirect				
Total	–1.45*	–1.91	–0.99	27.3%
Emotional warmth	–0.19	–0.40	0.02	
Rejection	–1.08*	–1.45	–0.73	
Overprotection	–0.18*	–0.31	–0.07	
**Maternal IPV on total scale score**				
Total	–4.74*	–5.85	–3.62	
Direct	–3.29*	–4.34	–2.24	69.4%
Indirect				
Total	–1.45*	–1.91	–1.02	30.6%
Emotional warmth	–0.13	–0.28	0.02	
Rejection	–1.12*	–1.51	–0.78	
Overprotection	–0.20*	–0.35	–0.08	

IPV, intimate partner violence; HRQOL, health-related quality of life. (Adjusted for children’ age, gender, status of single child, sleep duration, primary caregiver, maternal age, current marital status, educational level, and monthly per-capita income). ^a^The proportion of the total effect. *p < 0.05.

## Discussion

In this cross-sectional study, approximately 7.4% of the mothers have experienced IPV in their lifetime. Preschool children of mothers with IPV had significantly lower scores in all dimensions and summary scales of PedsQL 4.0 measured HRQOL, compared to their counterparts whose mothers reported no experience of IPV. In addition, we found that the associations between maternal IPV and children’s physical, psychosocial, and overall HRQOL were mediated by maternal parenting styles of rejection and overprotection.

Our findings indicated that maternal IPV was significantly associated with children’s adverse health outcomes, which was supported by previous studies (13, 39, 40). A cross-sectional study conducted in Japan has revealed a significant association between maternal IPV and poorer HRQOL in their preschool children (13). A cohort study conducted in Brazil has also shown that children of mothers with exposure to IPV had increased risk of behavioral and emotional difficulties, compared to those of mothers without experience of IPV (39). Furthermore, in an investigation conducted in India, maternal IPV has been found to be a risk factor for acute respiratory infection (aOR: 1.93, 95% CI: 1.42–2.61), diarrhea (aOR: 2.08, 95% CI: 1.74–2.46), and fever (aOR: 1.82, 95% CI: 1.55–2.14) in children aged 5 years or younger (40).

Furthermore, we have shown that maternal emotional warmth was positively associated with children’s psychosocial health, while maternal parenting styles of rejection and overprotection were negatively associated with children’s physical and psychosocial health. These results were supported by recent evidence about the superiority of high parental warmth and low strictness in children’s psychosocial adjustment (24, 41–44). For example, a cross-sectional study with participants from Spain, the United States, Germany, and Brazil, has found that indulgent parenting style (i.e., high parental warmth and low strictness) was associated with greatest personal and social wellbeing in adolescents (41). Another cross-sectional study conducted in Spain has also shown a significant link between parental warmth and better adolescent adjustment, while the opposite was the case for parental strictness and adolescent adjustment (42). However, several studies conducted in American families have identified that only the combination of parental warmth and strictness together (i.e., authoritative parenting) could promote adolescents’ development and socialization (25, 45, 46). In addition, strict parenting style (i.e., authoritarian parenting) has been found to be beneficial to children’s psychosocial adjustment and academic success in Chinese Americans (47), African Americans (48), and Arab societies (49). Nevertheless, a recent longitudinal study conducted in mainland China has recognized the superiority of authoritative parenting style in children’s adjustment, including social competence, academic achievement, self-esteem, mother-child relationship, and behaviors (50). Such inconsistent findings indicate that the influence of parenting styles on children’s psychosocial adjustment and socialization is different by society and culture (24, 51), and is depending on the age of children involved (21).

The current study also indicated the mediating role of parenting styles in the association between maternal IPV and children’s HRQOL, which was in line with previous studies (52–56). For example, a cross-sectional study of preschool children has suggested harsh parenting style as a mediator in the association between maternal psychological IPV and children’s disruptive behaviors (52). A longitudinal study of 905 mothers and their children has found aggressive parenting behaviors (i.e., spanking) to be a mediator in the association between maternal IPV and children’s poor mental health and social relationship at the age of 5 years (53). Another prospective cohort study conducted in the United States has also indicated that maternal IPV was significantly associated with lower levels of maternal sensitive parenting, which could subsequently impair the cognitive development in their children (54). Furthermore, a study based on a cohort of 308 mother-child dyads has shown that preschoolers of mothers with psychological IPV exposure tended to have more mental problems, and this association was partially mediated by restrictive and punitive parenting strategies (55). However, a cross-sectional study of 230 African American mothers and their children has failed to show a significant mediating effect of parenting styles in the association between maternal violence exposure and children’s behavioral problems (57). The inconsistent findings suggested the need for further studies to confirm the mediating role of maternal parenting styles.

The mechanisms of the association between maternal IPV and children’s adverse health outcomes remain unclear. Several hypotheses have been proposed by previous studies to explain such association. First, exposure to maternal IPV has been demonstrated to be detrimental to the emotional and neurological development in young children, who rely on their mothers to provide a safe and predictable environment where they can explore (58). However, when a mother herself was involved in IPV with stress, she usually failed to provide protection and care to her dependent children, which in turn could damage children’s brain development in related domain and resulted in adverse health outcomes and reduced HRQOL (12, 59). Second, maternal IPV usually cooccurs with child maltreatment in the same family (60, 61). Children of mothers who were exposed to IPV had increased risk of emotional abuse, physical abuse, and neglect (60). This violent family climate could impede children’s development, leading to various physical and mental health issues, as well as poor HRQOL (62, 63). Third, parental stress has been demonstrated to be a significant predictor of poor psychosocial adjustment, leading to increased risk of mental dysfunction, such as depression, anxiety, and stress, in parents (14). Thus, as a highly stressful situation, suffering violence from an intimate partner could impair psychosocial adjustment in mothers (5, 64), which could interfere children’s normal development (65, 66) and was associated with increased risk of children’s psychosocial and behavioral problems (66), subsequently leading to reduced HRQOL. Last, maternal IPV experience has been found to be associated with less effective parenting skills and engagement, as well as more neglect and aggression (28), which might lead to more behavioral and psychological problems in young children (55, 67). Therefore, the significant association between maternal IPV and poorer HRQOL observed in our study was plausible.

Our findings were consistent with previous evidence that family variables, such as background, family structure, and parenting stress, have great impact on children’s development and adolescent adjustment (68–70). For example, a cross-sectional study of 643 Portuguese adolescents have found that students from native families were more engaged in schools than those from immigrant families (68). Another cross-sectional study of 865 American adolescents has shown a significant link between family structure and behavioral problems, demonstrating that adolescents living with both natural parents were less likely to engage in deviant behaviors than those with single parent or stepparents (69). The present study further extends the findings of previous research by showing that even though maternal IPV negatively affects mothers and their children, parenting styles still have independent impact on children’s wellbeing. These findings indicated that interventions targeting at parental support and rearing styles might have substantial benefit to the wellbeing of vulnerable children and adolescents, and even to their later-life health outcomes (71, 72).

A major strength of this study was the usage of HRQOL as the outcome to fully reflect children’s physical, psychosocial, and overall health. We have obtained sufficient mother-child dyads to reinforce the statistical power. Furthermore, we have adjusted for several available confounders in the regression models. The mediating role of parenting styles were also evaluated, which provided hints for intervention target. However, several drawbacks of this study should be considered as well. First, the cross-sectional design precludes us from concluding any causal relationship. Further longitudinal research is needed to elucidate the temporal associations. Second, all preschool children were included only from one megacity in China, which might cause selection bias. The representativeness of this study should be interpreted with caution. Third, we were unable to determine whether the timing and duration of IPV experienced by mothers had different impacts on their children’s HRQOL (73, 74). Fourth, due to the young age of preschool children, HRQOL was measured with parent-proxy reports, rather than a child’s self-perception, which is a common limitation faced by child health researchers (12, 75). Last, although we have adjusted for several confounders in the multivariate analysis, some reported risk factors of HRQOL were not included due to data unavailability (12, 75).

## Conclusion

Preschool children whose mothers were victims of IPV had significantly poorer HRQOL compared to those of mothers without IPV experience. The associations between maternal IPV and children’s HRQOL were mediated by maternal rejection and overprotection. Child care workers (e.g., doctors, teachers, and social workers) should pay special attention to the wellbeing of children who are living in families with maternal IPV. In addition, parenting support should be provided to mothers with experience of IPV, in order to preserve their children’s HRQOL.

## Data availability statement

The original contributions presented in this study are included in the article/supplementary material, further inquiries can be directed to the corresponding author/s.

## Ethics statement

The studies involving human participants were reviewed and approved by the Ethics Committee of School of Public Health, Sun Yat-sen University (Ethical approval number: 2021[116]). Written informed consent to participate in this study was provided by the participants or their legal guardian/next of kin.

## Author contributions

VG: conceptualization. SL and VG: methodology and writing—original draft preparation. LL, CL, and VG: data curation. SL and LL: formal analysis and investigation. CL and VG: funding acquisition. All authors contributed to the writing—review and editing, read, and approved the final manuscript.
